# *Aedes aegypti* reproductive aspects: constant light significantly affects the embryonic development

**DOI:** 10.1590/0074-02760240233

**Published:** 2025-03-31

**Authors:** Luana Cristina Farnesi, Gabrielle da Silva Oliveira Alves, Luciana Ordunha Araripe, Rafaela Vieira Bruno

**Affiliations:** 1 Fundação Oswaldo Cruz-Fiocruz, Instituto Oswaldo Cruz, Laboratório de Biologia Molecular de Insetos, Rio de Janeiro, RJ, Brasil; 2 Fundação Oswaldo Cruz-Fiocruz, Instituto Oswaldo Cruz, Laboratório de Doenças Parasitárias, Rio de Janeiro, RJ, Brasil; 3 Universidade Federal do Rio de Janeiro, Instituto de Microbiologia Paulo de Góes, Laboratório de Bacteriologia Molecular e Marinha, Rio de Janeiro, RJ, Brasil; 4 Instituto Nacional de Ciência e Tecnologia em Entomologia Molecular, Rio de Janeiro, RJ, Brasil

**Keywords:** oviposition, arboviruses, fertility, Aedes aegypti, biological clocks, embryo development

## Abstract

**BACKGROUND:**

The importance of the mosquito *Aedes aegypti* as a vector of arboviruses like dengue, Zika, and chikungunya justifies the interest in investigating this species’ physiology and reproductive biology. For the maintenance and expansion of *Ae. aegypti* populations, copulation, oogenesis, female oviposition capacity, embryo development and larval hatching are crucial processes regulated by biological clocks. Many of these parameters have currently been investigated under environmental and laboratory conditions. However, there are specific gaps regarding the effect of light on these critical reproductive aspects. In this study, the influence of light on some aspects of *Ae. aegypti* biology was evaluated.

**OBJECTIVES:**

We investigated, in laboratory conditions, the effects of constant light on *Ae. aegypti* reproductive features: spermathecal content, embryo morphology, females’ fecundity, and egg viability.

**METHODS:**

Morphological and physiological assays were performed using *Ae. aegypti* females and eggs obtained from forced egg laying. The reproductive aspects were analysed under constant light (LL = light/light) and light/dark cycles (LD12:12 = 12 h of light and 12 h of dark).

**FINDINGS and MAIN CONCLUSIONS:**

Our results proved the negative effect of constant light on egg production (decreasing the fecundity) and embryonic development (causing a drop in egg viability and perceptive damage in the embryos). The results presented here bring new information on the impacts that a source of constant light may have on the reproductive biology of *Ae. aegypti.*

*Aedes aegypti* mosquitoes are vectors of essential arboviruses such as dengue, Zika, and chikungunya. This mosquito has populations distributed across the tropical areas of the globe, mainly in places where climatic conditions are more suitable for maintaining its biological cycle, like tropical and subtropical regions.^1^ Much of the human population lives in these areas, where humidity and temperature are high, which leads to a high risk of arboviruses transmission, for which *Ae. aegypti* is one of the main vectors.^1^ ^,^ ^2^

Several aspects related to the biology of insects are known to be crucial for the maintenance of populations of vector insects, such as *Ae. aegypti*.^3^ Among them, feeding, mating, oviposition, fecundity, and fertility may show circadian patterns, and the light/dark cycle is a crucial condition to maintain these aspects efficiently.^4^ ^-^ ^10^

The functioning of the endogenous circadian clock (central and peripheral) is synchronised by several environmental factors, among which the most important is light.^11^ However, it is worth noting that an alternation between light and dark phases in a 24 h cycle is fundamental to the clock entrainment. Rivas et al.^12^ demonstrated that the circadian expression of the central clock genes in the head is abolished when these insects are exposed to constant light for a few days, which leads to arrhythmicity in locomotor activity. Thus, once the clock gene expression is abolished in the central clock, exposure to constant light may disrupt other processes in the mosquito. Along with these results, Farnesi and collaborators^8^ showed a critical decrease in egg viability when females were exposed for three days to constant light, while the light/dark cycle was associated with greater egg viability.

There is currently a vast amount of knowledge about the reproductive behaviour of *Ae. aegypti*, including insemination, embryonic development, egg characteristics, and oviposition aspects.^3^ ^,^ ^4^ ^,^ ^8^ ^,^ ^13^ ^-^ ^17^ Briefly, after copulation, females permanently store sperm in structures called spermathecae. *Ae. aegypti* mosquitoes have three spermathecae in the female reproductive system. In the ovaries of blood-fed females, oocytes migrate to the lateral oviducts as they mature and are fertilised only when they pass through the central oviduct, connected to the spermatheca, shortly before laying.^18^ ^,^ ^19^ It is after laying that embryonic development begins. The number of full spermatheca after copula is usually two or three. A previous study with *Ae. aegypti* reared in the laboratory and allowed to copulate freely in cages showed that only 8% of females stored sperm in two spermathecae, while 92% stored in all three spermathecae.^19^

The embryonic development of *Ae. aegypti* can be followed by the morphology of the embryos, visible after chemical clarification of the eggshell, at certain stages when the main morphological landmarks of embryogenesis occur in constant temperature.^15^ ^,^ ^20^ ^,^ ^21^ Thus, it is possible to know if an *Ae. aegypti* embryo is still viable inside the egg. An easily visible morphological landmark is the extension and retraction of the germ band that occurs in the first third of embryogenesis, about 24 h after egg laying (at 28*º*C).^21^ In addition, at the end of embryonic development (61.6 h ±1.2 at 28*º*C), with the clarified eggshell, it is possible to visualise the larva completely formed inside the egg: the head, the fused thoracic segments and the abdominal segments being easily recognisable.^15^ *Ae. aegypti* females can produce approximately 100 eggs in each gonotrophic cycle (interval between blood meal and oviposition). The production of eggs by females is called fecundity and is an essential parameter of vector capacity.^4^ ^,^ ^6^ ^,^ ^10^

Given the importance of this mosquito vector, there are still some gaps in the knowledge about *Ae. aegypti* reproduction behaviour, such as the timing of its development under constant light. Here, we intend to investigate if the constant exposition to light (i) prevents the sperm transfer to females, (ii) prevents copulation, (iii) changes the number of spermathecae with spermatozoa, (iv) alters fertilisation, and (v) prevents the formation of a viable embryo.

## MATERIALS AND METHODS

*Mosquito rearing* - Mosquito eggs were provided by the Laboratório de Biologia, Controle e Vigilância de Insetos Vetores (LBCVIV), Instituto Oswaldo Cruz, Fiocruz, Rio de Janeiro. *Ae. aegypti* of the Rockefeller lineage^22^ were used in all experiments. They were reared and kept in the Laboratório de Biologia Molecular de Insetos (LABIMI) insectarium in an incubator (Forlab Scientific Incubator, USA). According to Farnesi et al.,^15^ the mosquito eggs were hatched. After hatching, the first instar larvae were counted and distributed to plastic trays (500 larvae per tray containing 1.5 L of dechlorinated water) and fed with 1.5 g of Tetramin (Tetramarine Saltwater Granules, Tetra GmbH, Germany) every two days, until pupae full development. Pupae were counted and separated in cages (with approximately 150 males and 150 females) for adult emergence. Adult mosquitoes were transferred to cages that were placed in an incubator under the specific light regimen for each test: 12 h of light and 12 h of dark (LD 12:12) or constant light (LL) at 25 ± 1ºC and relative humidity between 60-80%. Male and female mosquitoes were kept in the same cage (feeding on 10% sucrose solution *ad libitum*) to allow copulation for at least three days.

For all tests, we considered: Day 0 = day adults emerged (metamorphosis from the pupa stage to the adult stage); Day 3 = sugar-deprived females (4 h with no food) were taken to blood-feed on anesthetised Swiss mice; Day 7 = synchronous egg laying induction.

*Spermatheca assays* - The insemination status was checked in females not blood-fed, on Day 3, for different light conditions. We removed the last two abdominal segments with the three spermathecae internally attached and placed them on a glass slide. A volume of 10 uL of sterile phosphate buffered saline (PBS) (sodium phosphate buffer 0.1 M) was added to the slide and used as a dissection medium. Spermathecal reservoirs were broken using entomological forceps. We considered positive the spermatheca that had sperm content visible under the Axioskop 40 stereo microscope (Zeiss) and negative spermatheca those where sperm was not visible. Three experiments were performed with 40 females per light condition. A total of 240 *Ae. aegypti* females were analysed.

*Fecundity and fertility assays* - We performed three experiments per light condition; each one contained at least 50 females. On Day 3, the female mosquitos were deprived of sugar before the blood meal on anesthetised Swiss mice (Comissão de *Ética* de uso de animais - CEUA-FIOCRUZ LW-28/18) for approximately 4 h. In all fecundity and fertility assays, fully engorged female mosquitoes were selected. After four days (on Day 7), the females were induced to synchronised oviposition (according to Farnesi et al., with minor changes).^15^ In summary, females were individualised in inverted Petri dishes (90 mm in diameter) and with the lid lined with filter paper (Whatman nº 1). After transferring one female per plate, with a Pasteur pipette, 3 mL of basin water (distilled water and yeast) was added to the plate lid to entirely wet the filter paper, stimulating the female›s oviposition.

The females of both groups (LD12:12 and LL) were left in separate plates for 90 min inside incubators with the tested light condition, with constant temperature (25 ± 1ºC) and relative humidity of 60-80%. At the end of oviposition time, the females were discarded, and the eggs were kept in the same incubators until the end of embryogenesis (minimum of 77.5 h, as described in Farnesi et al.^15^ in a humid environment, Day 10). After this time, the eggs remained in the dry for a maximum of seven days to avoid any changes in viability (day 17). Fecundity is the number of eggs laid per female. The eggs were visually counted with a *Tigre* brush nº 308-0. Fertility analyses (egg viability) were performed individually on each plate with eggs. All eggs from all females of fecundity experiments were hatched as follows: 50 mL of industrial yeast extract 0.15% (weight/volume) were added to each plate to stimulate hatching by the presence of organic matter.^15^ Then, these plates were placed in incubators for 24 h (25 ± 1ºC, relative humidity of 60-80%) under LD or LL regimens. After this time, the first instar larvae from each plate were counted, and the percentage (from each female) was calculated.

*Embryo morphology analysis* - In order to analyse the embryo morphology, ten females were induced to lay eggs on day 7, as described above. Eggs were kept moist until the end of embryonic development.^15^ All eggs from each light condition (LL or LD12:12) were exposed to Trpiš’s solution after embryonic development (77,5 h after egg laying, at 25ºC). This procedure is for eggs to be fixed and clarified,^20^ which makes the eggshell transparent and allows the identification of embryo morphological status. Embryos inside the resulting transparent eggshells were observed under an Axioskop 40 microscope (Zeiss) and photographed in a Stereo Discovery V.12 stereoscope (Zeiss). Final embryonic stages and embryo viable aspect were identified in compliance with previous references.^15^ ^,^ ^21^

*Statistical analyses* - In all analyses of reproductive parameters, we first performed the Shapiro-Wilk normality test. Pairwise comparisons of the percentage of positive or negative spermatheca of females exposed to different light regimes (LL X LD) were performed with the Kruskal Wallis test followed by the Dunns multiple comparison test. Mann-Whitney tests were used to compare fecundity (number of eggs per female) and viability (percentage of hatched eggs). All statistical analyses and graphics representation were performed with the GraphPad Prism 5 software (Graphpad Software, Inc.).

*Ethics statement* - The procedures performed in this study were approved by the Animal Ethics Committee (protocol LW-28/18) of Fundação Oswaldo Cruz (Fiocruz).

## RESULTS

**Fig. 1:**
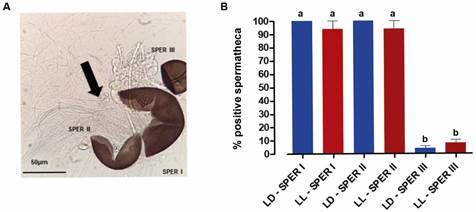
sperm is present in spermathecae of *Aedes aegypti* regardless of light regimen. (A) Ruptured spermathecae from inseminated *Ae. aegypti* females. The three spermathecae are shown in this photo: one large (Sperm I) and two smaller (Sperm II and Sperm III). The arrow points to sperm. Bars = 50 µm. (B) Percentage of the positive spermatheca, that is, spermatheca with visible sperm, from females of each regimen. According to Kruskal Wallis with Dunns multiple comparison test, letters represent the statistical difference among the groups.

*Constant light does not interfere with copulation or sperm transfer* -For the two light conditions, sperms were found in at least two of the three spermathecae of all females analysed after three days in contact with males, always in the larger spermathecae (Sperm I) and one of the smaller (Sperm II) (Fig. 1A). It was possible to detect male gametes because they are like thin translucent threads (Fig. 1B). In both light regimens, spermathecae were all positive in only 10% of the analysed females (Fig. 1A). There was no significant difference between the analysed light regimens. However, in all cases, the number of positive spermathecae I and II were significantly different from those of spermathecae III (KW = 35.78, p < 0.0001).

**Fig. 2:**
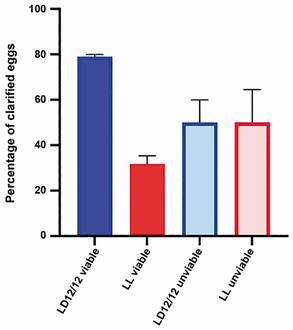
egg viability verified by the morphological aspect of the embryo. Synchronised eggs exposed to light and dark regimen (LD) (A) or constant light (LL) (B) conditions at the end of embryogenesis were clarified. Data are expressed as the percentage of viable and unviable eggs for each regimen. Each bar represents the median of three experimental replicates and the 95% confidence interval (CI).

**Fig. 3:**
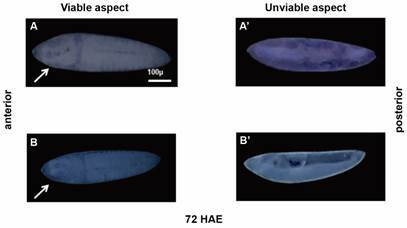
morphology of the *Aedes aegypti* embryo at the end of embryonic development is modified by exposure to constant light. In A and A’, embryos kept in light/dark cycles; in B and B’, embryos kept in constant light. Images obtained after clarification of the shell and fixation of the embryo (see Methods). Arrows: embryo head region. Bars: 100 µm.

*Constant light interferes with the embryo morphology* - Eggs from females exposed to constant light (LL) or a light and dark regimen (LD) were kept under these conditions until the end of embryonic development.^15^ Afterwards, all eggs were clarified.^20^ Most eggs kept in LD (median of 79%) showed a viable and fully formed embryo ready to hatch (Figs 2, 3A, to compare with 3A’). On the other hand, fewer eggs (a median of 31.7%) maintained in LL showed the appearance of viable eggs; that is, most embryos did not develop well and died (Figs 2, 3B, to compare with 3B’).

**Fig. 4:**
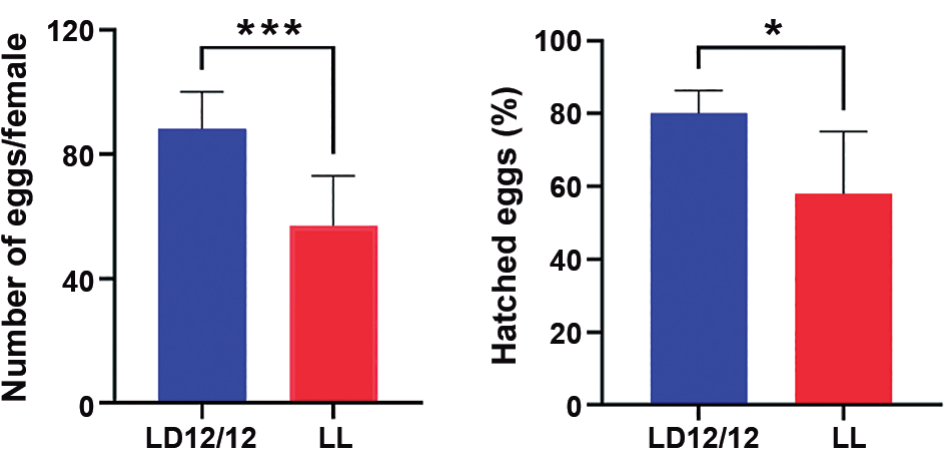
comparison of fecundity and fertility after the first gonotrophic cycle between females of *Aedes aegypti* from light and dark regimen (LD) 12:12 and constant light (LL). (A) Columns represent the medians of the number of eggs laid, and bars represent the 95% confidence interval (CI). In blue is the LD regimen, and in red is the LL regimen. Significance is represented by asterisks (p < 0.001 obtained by the non-parametric Mann-Whitney test). (B) Columns are the medians of the percentage of eggs hatched from each regimen, and bars represent the 95% CI. In blue is the LD regimen, and in red is the LL regimen. Significance is represented by asterisks (p < 0.05 obtained by the non-parametric Mann-Whitney test).

*Fecundity and fertility decrease in constant light* - In order to investigate whether constant light affects *Ae. aegypti’s* fecundity (egg production) and fertility (egg viability) on the first gonotrophic cycle, we comparatively analysed eggs originated from females exposed to LL (and maintained in the same light regimen) *versus* eggs from females exposed to a light-dark (LD12:12) cycle (and maintained in the same light regimen). The results of comparing the number of eggs per female between LL and LD light regimes are shown in Fig. 4A. The fecundity was significantly higher in the LD regime (median of eggs per female = 88 LD and 57 LL). The constant light significantly affected this reproductive aspect (U = 655.0; p < 0.001). Moreover, the viability of these eggs also decreases significantly in the LL regimen compared to the LD regimen (80% LD and 58% LL; U = 770, p < 0.05, p < 0.05, Fig. 4B).

## DISCUSSION

Within the Culicinae subfamily, mosquitoes of the *Aedes* genus are the main vectors of arboviruses that cause human diseases, such as yellow fever, dengue, Zika, and chikungunya.^1^ ^,^ ^18^ ^,^ ^23^ ^,^ ^24^ ^,^ ^25^ The *Ae. aegypti* mosquito is considered the primary vector of the dengue virus and is involved in the transmission of other important arboviruses.^26^ ^,^ ^27^ ^,^ ^28^ Some characteristics of *Ae. aegypti* reproductive biology are related to its geographic expansion over the years, making it difficult to control its populations.^28^ ^,^ ^29^ Among them are: (i) a single insemination event to ensure the supply of sperm in the spermathecae to fertilise all the eggs that a female will develop during its lifetime; (ii) the habit of spreading the eggs over many breeding sites, (iii) the diversity of egg-laying sites and (iv) the capacity of the eggs of this species to remain viable in the dry environment, quiescent, for long periods, waiting for the breeding site to flood again.^3^ ^,^ ^14^ ^,^ ^30^ ^,^ ^31^ ^,^ ^32^ ^,^ ^33^ Despite the importance of reproductive characteristics and the relevance of *Ae. aegypti* as an arbovirus vector, there are still significant gaps in many of these aspects, especially regarding exposure to light and temperature cycles in a changing environment. For instance, little is known about the effects of constant light conditions, similar to what is generated by artificial lighting in the urban environment, on the reproductive biology of this species. Farnesi and collaborators have previously analysed the fecundity and fertility of females exposed to constant light compared to females exposed to LD or DD regimens.^8^ Here, we investigated if male exposure to constant light can interfere with decreased egg production.

In mosquitoes, insemination is defined as the deposition of semen (sperm and secretions from the male accessory glands) into the female’s pouch and the subsequent passage of the sperm into the spermatheca.^4^ In the Culicinae subfamily, the number of spermathecae varies between one and three. In the species *Ae. aegypti*, three spermathecae are found, one large and two smaller.^3^ ^,^ ^34^ The ultrastructure of *Ae. aegypti* spermatheca has been previously described;^3^ this compartment is a sclerotised, spherical, and chitinous structure composed of a round, dark brown reservoir, connected to the bursa copulatrix by a translucent spermathecal duct. After insemination, spermatozoa are arranged circularly inside the lumens of the large and small spermatheca reservoir.^3^ ^,^ ^35^ ^,^ ^36^ Determining insemination status is critical for investigating behavioural and molecular interactions between males and females. There are several methods to determine the status of insemination.^36^ ^,^ ^37^ However, verifying whether a culicine spermatheca contains sperm is relatively easy with light microscopy assays; if sperm are present, it is possible to see a mass of threads in the spermathecae.^37^ ^,^ ^38^ The *Ae. aegypti* sperm remains viable in the spermathecae for the whole life of the female.^3^ ^,^ ^36^

Here, in both light regimens, it was possible to see the sperm in circling movements in all positive spermathecae analysed (Fig. 1). Our results show at least two positive spermathecae (containing sperm) per female, which suggests that constant light did not interfere with male reproductive aspects since the result of insemination was not different between light regimens.

The viability of mosquito eggs is directly related to the development of embryos in the environment in which they were laid. In this sense, the resistance of eggs to environmental desiccation is strongly associated with the colonisation of Culicidae species in new geographic regions.^39^ The exposure of eggs to dry environments and the morphological characteristics of these embryos have already been widely studied. It is known that for the species *Ae. aegypti*, *Anopheles aquasalis*, *An. gambiae* and *Culex quinquefasciatus*, the formation of the serous cuticle (chitinous structure formed in the first third of embryonic development) is crucial.^15^ ^,^ ^40^ ^,^ ^41^ The morphological aspect of the mosquito embryos at the time of formation of the cutical serosal (CS) corresponds to the extension of the germinal band; this is easily recognised when the eggshell is clarified, and in light microscopy, the embryo is seen inside the egg.^15^ ^,^ ^20^ ^,^ ^21^ ^,^ ^40^ Here, we use previously described morphological features of *Ae. aegypti* embryos for characterising completed embryonic development as parameters for confirming viability.^15^ ^,^ ^21^

As described by Farnesi and collaborators,^15^ it is possible to observe, at the end of embryonic development (~ 72 h at 25ºC), the larva fully formed inside the egg (the head, fused thoracic segments and abdominal segments are easily recognisable). Here, only 20% of the eggs exposed to the LL condition (originated by insemination with males also exposed to the LL condition) achieved this status (Figs 2-3). In comparison, it occurred in ~ 80% of the eggs exposed to the LD cycle. Thus, when we compared the embryos clarified in the two conditions (LL versus LD), we confirmed the interference of exposure to constant light on the morphological formation of embryos at the end of embryogenesis (Fig. 3). Studies in *Ae. albopictus* relate the embryos’ formation time and their morphological stage (using an eggshell clarification technique) with the hours of light to which they are exposed: a regimen of 8 h of light and 16 h of dark (LD 8:16) is enough to induce photoperiodic diapause. Therefore, embryos complete their formation when exposed to photoperiods longer than 16 h of light and 8 h of dark, proving the importance of light/dark cycles for the *Aedes* genus.^42^

Due to the importance of *Ae. aegypti* as a vector of several arboviruses, fitness studies require an evaluation of parameters such as egg production per female and hatching rates at the individual level (viability). Agudelo and collaborators^43^ tested the influence of male age on fecundity and fertility. They observed that this factor had no significant effect on these reproductive parameters, showing that, in addition to sperm transfer commonly occurring with young and old males, posture and embryogenesis commonly occur.

Here, we studied the effect of an abiotic agent (constant light) on the reproductive aspects of *Ae. aegypti.* For the first time, we show that constant light significantly influenced the number of eggs laid per female (fecundity) mated with exposed males. On the other hand, the difference in egg viability (fertility) in LD compared to LL condition corroborates what our group previously described as a tendency: regardless of the preference of *Ae. aegypti* females for laying eggs in dark sites, the light and dark cycle is fundamental for the eggs to be mostly viable.^8^ In this same work, the authors showed that *Ae. aegypti* females kept in a constant light (LL) regime for 84 h (three days and 12 h) laid a number of eggs not significantly different than females kept in the light-dark cycle (LD) for the same time. However, when the oviposition stimulus was given at 96 h (four days), the number of eggs per female was reduced per female exposed to constant light.

Furthermore, although the equivalence in the number of eggs laid in LL or LD in 84 h of treatment in both conditions, when compared to the eggs’ viability, a significant reduction was noticed for the eggs of females kept in a constant light regimen. Despite knowing the negative effect of light on these parameters, our group had not yet been able to state in which specific reproductive step the light was interfering, whether in the copulation and sperm transfer phase or in the embryo’s development.^8^ Here, with the light microscopy observation for the presence of sperm in the spermathecae, we conclude that constant light did not affect copulation or sperm transfer. Instead, the clarification egg assays that allowed visualisation of the morphology of viable and non-viable embryos showed that constant light interfered with the viability and morphology of these embryos by a still unknown mechanism.

Natural light/dark cycles have been suffering alterations in an enormous part of the globe, thanks to the fast growth of artificial and efficient lighting (LED) in urban and suburban environments. The effects of this anthropic disturbance are known for several species of insects and other organisms,^44^ ^,^ ^45^ ^,^ ^46^ but not much investigated for *Ae. aegypti* ^47^ and other mosquito vectors.^48^ ^,^ ^49^ ^,^ ^50^ Although Artificial Light at Night (ALAN), also known as light pollution, is a severe factor affecting organisms and trophic interactions,^51^ it must not be related to the LL condition set in the lab incubator. Instead, the constant light treatment implies the maintenance of light intensity throughout the whole period, which disrupts the circadian rhythm of expression of several clock genes, imposing a high physiological challenge to the organism.^12^ In this sense, our results must be interpreted as the steps of *Ae. aegypti* reproduction that may be responding to the physiological disruption imposed by the constant light.

Our results strongly suggest that the constant light has a negative effect on oogenesis (decreasing the fecundity) and embryonic development (causing a drop in egg viability and perceptive damage in the embryos). The results may clarify future studies on the reproductive biology of this important vector of arboviruses, the mosquito *Ae. aegypti*. Further investment in identifying additional traits influenced by the disruption of light/dark cycles should provide important information about this species’ biology, generating bases to develop new control possibilities for this important vector.
